# The Hospital Sunday Fund

**Published:** 1892-02-06

**Authors:** 


### THE HOSPITAL SUNDAY FUND.

A MEETING of the Council of the Metropolitan Hospital Sunday Fund was held at the Mansion House on the 1st inst. In the absence of the Lord Mayor, Archdeacon SINCLAIR presided.—The SECRETARY (Mr. Henry W. Custance) announced a donation of £500 from "E. A. H.," and a donation of £50 from "E. H. F."—Sir Rutherford Alcock having sent in his resignation as a member of the Council owing to advanced age, the Rev. Dr. MARKS moved, and Mr. BURDETT seconded, a vote of thanks to Sir Rutherford for his past services, of which the Council entertained a pleasant recollection. The resolution was passed.—On the motion of Mr. W. R. TIDD, seconded by Dr. W. SEDGWICK SAUNDERS, a vote of condolence was sent to the family of the late Sir J. Risdon Bennett, who had served on the Council for several years.—Another member of the Council whose death was announced by the Secretary was Cardinal Manning.—The Rev. Dr. KENNEDY moved that a vote of condolence should be sent to the representatives of the late Cardinal through the Vicar-General. The late Archbishop of Westminster, he said, had devoted himself to the work of the fund with hearty and intelligent interest. That interest he continued to show as long as his health allowed.—The CHIEF RABBI (Dr. Adler) seconded the motion, and it was carried.—Vacancies caused by death and resignation on the General Purposes Committee were filled, the Chief Rabbi taking the place of Cardinal Manning, Lord Sandhurst that of Mr. R. Capel, Mr. Wakley that of Sir Rutherford Alcock, Mr. A. L. Cohen that of Mr. J. Boodle. Dr. Hare that of Mr. Francis Hoare, and Canon Ingram that of Mr. T. Sadler.

Mr. BURDETT moved the adoption of a uniform system of accounts for hospitals and dispensaries. Each institution had previously kept its accounts according to its own plan, so that there were insurmountable difficulties in the way of comparisons. The proposed scheme was not an attempt at reform, but was an accomplished fact, hospitals connected with the fund having agreed to its adoption. The measure of abuse that had existed might be gathered from the knowledge that in some cases £200 had been spent in order to raise £100. At present nothing of this kind could be found in connection with any metropolitan hospital worthy of the name, and certainly not in connection with any hospital associated with the Sunday Fund.—Dr. GLOVER seconded the motion.—Mr. ACLAND urged that there should be an item showing workpeople's contributions other than from the Saturday Fund.—Dr. GLOVER condemned church parades as noisy and disagreeable, and out of all proportion to the income which was derived from that source.—Mr. BURDETT suggested that on the *Income* side of the account, after *Hospital Saturday Fund*, there should be a second heading, Va.—*Workpeople's Contributions (not included in Hospital Saturday)*, (1) from workshops, and (2) from Church parades.—Dr. SAUNDERS, who took the chair on the departure of Archdeacon Sinclair, urged that the original plan which had been already approved by the secretaries of hospitals should be adopted, say, for one year. This would give them experience and would avoid delay. After some discussion, the form of accounts was adopted, and the suggestion of Mr. Acland was approved for further consideration.—At the close of the business, the Rev. Dr. RIGG proposed a vote of condolence with the widow of Mr. Spurgeon, who had greatly assisted the fund, and who was a great loss to the moral force of the country.—The CHIEF RABBI, in seconding the motion, remarked that though there might be no precedent for such a vote, it would be a graceful act towards one who had done great service.—Mr. BURDETT supported the motion, which was adopted, and reminded the Council that when the special effort was made to increase the fund by which it was raised from under £30,000 to £45,000 a-year Mr. Spurgeon had rendered very great assistance at considerable personal inconvenience.

